# Cancer Nanotechnology Startup Challenge: a new way to realize the fruits of innovation

**DOI:** 10.1186/s12645-016-0014-9

**Published:** 2016-03-04

**Authors:** Fred Currell, Mark Bellringer

**Affiliations:** Centre for Plasma Physics, School of Mathematics and Physics, Queen’s University, Belfast, UK; London, UK

## Abstract

A significant new innovation-development model is being launched in the field of cancer and nanotechnology. A significant new innovation-development model is being launched in the field of cancer and nanotechnology.

A significant new innovation-development model is being launched in the field of cancer and nanotechnology. Here at Springer’s *Cancer Nanotechnology* office, we thought it would be something of interest to our readers. Known as the *Nano Startup Challenge in Cancer*—or *NSC*^*2*^ for short—this exciting model is being run by the National Cancer Institute (NCI) in partnership with the Centre for Advancing Innovation and is being supported by MedImmune/AstraZenica. This article is the first in a set covering the challenge as it unfolds.

Following two award-winning competitions (the *Breast Cancer Startup Challenge* and the *Neuro Startup Challenge*) *NSC*^*2*^, the third in the series of challenges, is structured as an open innovation competition designed to bring promising nanotechnology cancer inventions to market.

Pivotal to the *NSC*^*2*^ challenge is a new business model: instead of potential costs of $2–5 M to secure an exclusive license leading to commercialization, a new startup exclusive license agreement may allow newly generated companies greater chances to bring inventions to market. Constructed by the National Institute of Health, the new license agreement will be the basis upon which the winning startups in the challenge go forward with upfront cost of only $2000 for an exclusive license agreement—details can be found at http://www.ott.nih.gov/nih-start-exclusive-license-agreements.

All of these inventions are concerned with nanotechnology as applied to cancer and all have the potential to make a significant therapeutic impact on the health of cancer patients.

In a notable difference from the two previous challenges, *NSC*^*2*^ inventions can originate from either the NIH intramural research program or the extramural research community. Entrants register via the website (http://www.nscsquared.org) and then self-assemble into teams which compete to take forward one of the inventions. Entry is open to anyone, anywhere in the world, provided they are over 18 years of age. The resulting multidisciplinary teams are required to have expertise in business, medicine, science and law. Each team will contain at least one serial entrepreneur and two students. The teams will interact with a panel of internationally renowned judges/mentors including heads of venture for large companies active in this sector.

As part of our coverage, we at *Cancer Nanotechnology* journal are going to offer teams the chance to write a scientific article about the invention they are progressing.

We interviewed Rosemary Truman, CEO for the Centre of Advancing Innovation, and Piotr Grodzinski, Director of National Cancer Institute Alliance for Nanotechnology in Cancer at the National Cancer Institute in Bethesda, to learn a bit more about the startup challenge model in general and this specific competition.

When asked about the value of engaging with the scientific community in this way, Rosemary Truman said that the teams would benefit from: “constructive feedback and exposure to people who think about it differently than they do. It may change their whole approach to commercialisation”. She sees the feedback and suggestions/guidance of the world-class industry and academic mentors and advisors as being immensely valuable as it draws from: “a large network of people thinking about the same kinds of problems.” In addition, the teams coming into the challenge are multidisciplinary and this allows scientists to engage with crucial talent that drive successful commercialization. Finally, the accelerator provides 40 classes that provide scientists an opportunity to learn the “business of science.”

Piotr Grodzinski was keen to emphasize the developmental and educational aspects of the process for those involved. He views the articles in *Cancer Nanotechnology* as an opportunity for teams to indicate: “why they think it can result in a practical clinical solution long term.” He also pointed out that the new paradigm could result in a new way of thinking for researchers, something he summarized with the pithy phrase: “Their sandbox becomes bigger.”

Of course, we will be watching and reporting on developments as the *NSC*^*2*^ challenge proceeds, as well as publishing the papers arising. If you feel you would like to enter the competition, it still is not too late; you have until 14th March—entry instructions along with other details are all available at http://www.nscsquared.org.

